# Dissecting cellulitis of the scalp: clinical characteristics and impact on quality of life of 66 Brazilian patients^☆^

**DOI:** 10.1016/j.abd.2024.06.003

**Published:** 2024-11-28

**Authors:** Paula Gerlero, Isabela Peron, Isabella Doche, Evelyn Freitas Rodrigues, Thalita Macedo, Maria Cecília Rivitti-Machado

**Affiliations:** Department of Dermatology, Hospital das Clínicas, Faculty of Medicine, Universidade de São Paulo, São Paulo, SP, Brazil

Dear Editor,

Dissecting cellulitis of the scalp (DCS), also known as “*perifolliculitis capitis abscedens et suffodiens*”, is a rare primary neutrophilic cicatricial alopecia, with a significant negative impact on the quality of life of patients.^1^ Along with hidradenitis suppurativa (HS), acne conglobata (AC), and pilonidal cyst (PC), DCS has been considered as part of the follicular occlusion tetrad, suggesting a common pathogenic mechanism for these entities.^2^, ^3^

DCS affects mainly African American males in the 2^nd^ and 3^rd^ decades of life; however, it has been described also in Caucasians^3^ and in females.^4^

The vertex and occipital areas of the scalp are predominantly affected. Initial lesions are comedonal-like structures with pustules, fluctuant nodules, and sterile abscesses in a patchy or diffuse pattern distribution of the scalp. In this early stage, trichoscopic features may be similar to alopecia areata (AA) with black dots, empty follicular openings, and double-bordered 3D-yellow dots; and early intervention in this stage can lead to regrowth hair^5^, ^6^ (Fig. 1). If left untreated, the lesions eventually result in inflammatory suppurative painful nodules and interconnecting sinus tracts that ultimately lead to scarring alopecia (Fig. 2).

**Figure 1:**
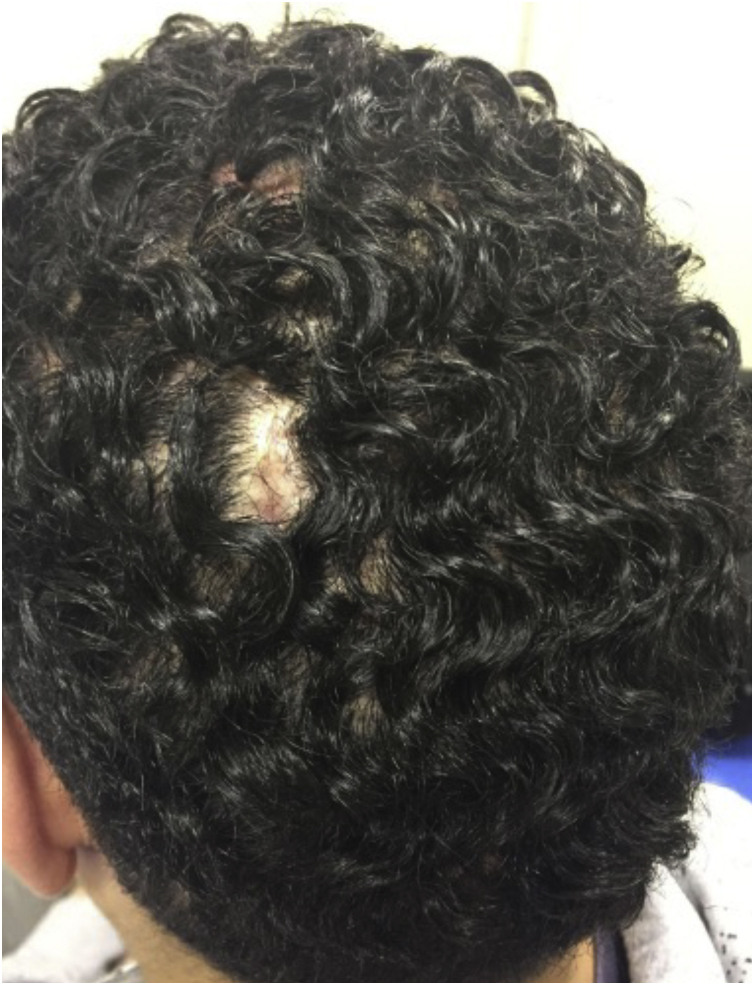
Dissecting cellulitis of the scalp: initial lesions are generally characterized by single nodules and non-scarring patches of alopecia.

**Figure 2:**
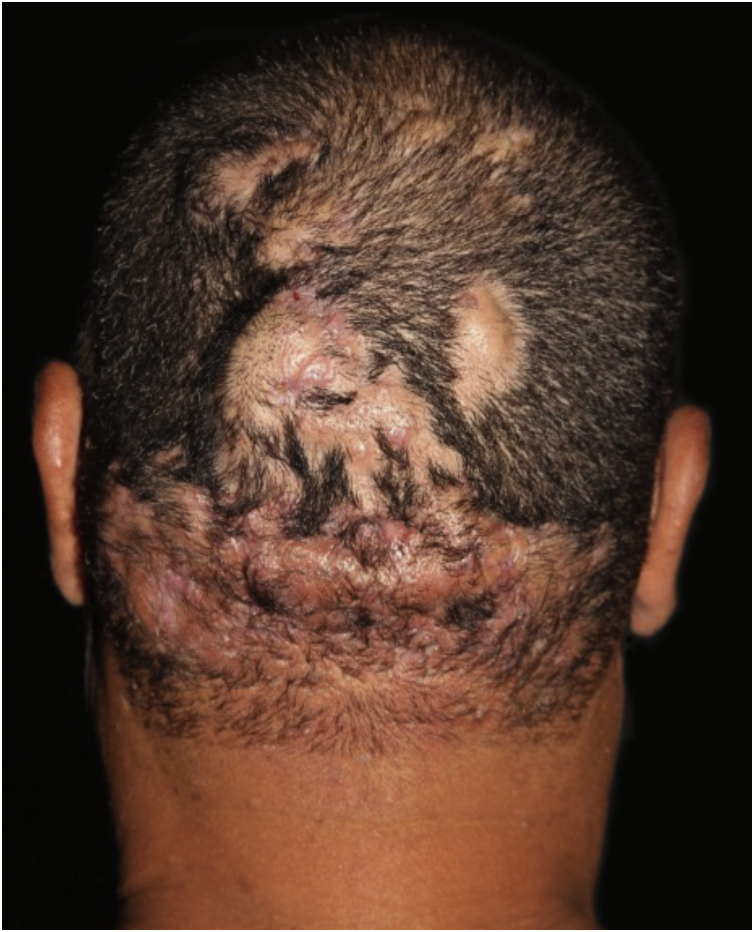
Dissecting cellulitis of the scalp: late-stage lesions present multiple inflammatory suppurative nodules, abscesses and interconnecting sinus with areas of scarring alopecia.

Diagnosis relies mainly on clinical and trichoscopy examinations, although histopathology can be useful in difficult cases.

As most lesions are chronic and may lead to areas of permanent alopecia, impairment in Quality of Life (QoL) has been reported in these patients.^7^

In this study, we describe demographics and impact on QoL of patients with DCS. This retrospective study included 66 patients (63 males, 3 females). All patients were diagnosed with DCS at the Dermatology Department of the Hospital das Clínicas of the University of São Paulo ‒ Brazil, between 2015 and 2023. Patients’ demographics were retrieved from medical charts. QoL was assessed using the Dermatology Life Quality Index (DLQI)^8^ questionnaire translated and validated for the Portuguese language. Exclusion criteria included associated alopecia and immunosuppression.

Our results showed a predominance of men (n = 63) with skin types III‒IV. The mean age was 30 years (14‒62); the mean Body Mass Index (BMI) was 28.40 (n = 23). All patients showed at least two of these features: areas of scarring alopecia, inflammatory nodules, abscesses, and sinuses over the scalp. Approximately 60% (n = 40) of the patients were treated with oral isotretinoin at a mean dose of 0.5‒1 mg/kg/day and 15% (n = 10) were treated with adalimumab 40 mg per week, with partial and mild response in both groups. Fifty-three percent (n = 32/60) of the patients with DSC presented comorbidities related to follicular occlusion tetrad with HS being the most frequent (n = 27, 45%), followed by AC (n = 21, 34.4%) and PC (n = 6, 10.2%) (Table 1). In the HS-DCS group, 44.4% (n = 12) were diagnosed with both diseases at the same time; in 28% (n = 7) DCS was first diagnosed and in 29.6% (n = 8) HS was first diagnosed. In the group of DCS first diagnosis the median time interval between disease diagnosis was 3 years (1‒7) and in the HS first diagnosis (n = 8) was 10 years (2‒22). DLQI was assessed in 47% (n = 31) of the patients. The mean DLQI score of these patients was 10, with scores ranging from 0 to 28.

**Table 1:** Population characteristics and quality of life in 66 patients with DCS.

Clinical Characteristics	N/Total	%
**Gender**		
Male	63/66	95.5
Female	3/66	4.5
**Fitzpatrick Skin type**		
I‒II	3/66	4.5
III‒IV	45/66	68.2
V‒VI	18/66	27.3
**Associated comorbidities**		
Hidradenitis suppurativa	27/60	45
Acne conglobata	21/61	34.4
Pilonidal cyst	6/59	10.2
Age^a^, years (mean)	14‒62 (30)	‒
DLQI score^a^ (mean)	0‒28 (10)	‒
DLQI score^a^ (mean) ‒ Follicular occlusion tetrad	10‒21 (17.5)	‒

N, Number of patients.

a Range.

In our study, the most affected DLQI domains were symptoms and personal relationships; no patients had a negative impact related to treatment, suggesting that impairment in self-image is more distressful than the treatment itself for these patients. Furthermore, QoL impairment was not influenced by sports or sexual life (Fig. 3). Patients with follicular occlusion tetrad (n = 5) showed a greater impact on QoL with a mean DLQI score of 17.5, compared with patients with no follicular occlusion tetrad.

**Figure 3:**
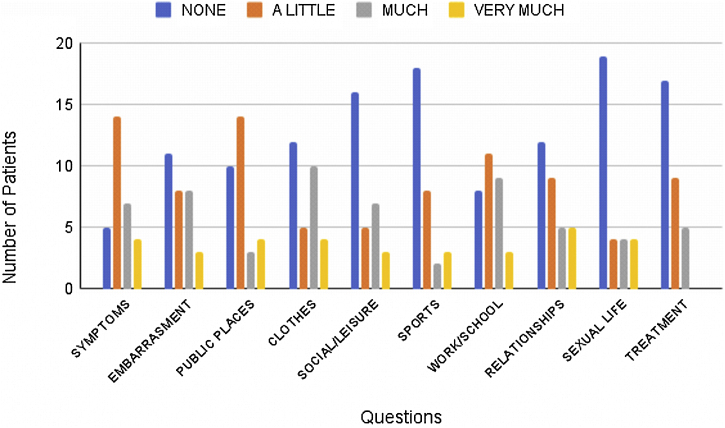
Frequency of responses to the DLQI questionnaire according to the questions. The DLQI consists of a self-administered questionnaire with ten questions concerning patients’ perception of the impact of the skin-hair disease on different domains of their health-related QoL over the last week. It covers six domains: symptoms and feelings (Q1, Q2), daily activities (Q3, Q4), leisure (Q5, Q6), work and school (Q7), personal relationships (Q8, Q9), and treatment (Q10). DLQI, Dermatology Life Quality Index, QoL, Quality of Life, Q, Question.

Pathogenesis of DCS remains poorly understood. The predominance in young men points to follicular occlusion as a key feature.^1^, ^5^, ^6^ However, a multicenter study reported a predominance of fair skin and Caucasian hair in women, suggesting a diverse pathomechanism involving immune dysregulation.^3^

The preponderance of the disease in younger patients and the association with HS in almost 45% are consistent with the literature.^1^, ^2^, ^3^, ^5^ In one study of more than 5000 patients with HS, DCS prevalence was 9.2%, compared to 0.7% in matched control individuals, and the risk of DCS in patients with HS was 13.38 times greater. These patients (DCS-HS) showed a greater impact on QoL compared with DCS alone.^9^

Previous studies have also shown that the reduced QoL in DCS patients is even more significant than in those with other inflammatory dermatoses, like psoriasis.^10^

As most lesions are located over visible scalp areas, patients tend to experience feelings of social rejection and stigmatization, with a great impairment in QoL, highlighting the necessity of early diagnosis and treatment.^10^

Treatment of DCS can be challenging. First-line therapy with oral isotretinoin, oral antibiotics, and intralesional steroids, are often used to control limited disease in milder presentations. Our study corroborated the efficacy of isotretinoin in the treatment of mild to moderate DSC.^6^ However, in the management of refractory cases or in association with HS, biologic drugs such as adalimumab, infliximab or the combination of biologics with other drugs, seems to be useful.^11^

Limitations of this study included retrospective design and that our results originated from a single academic center, and may not be generalizable.

It is important to measure the quality of life in patients with DSC, as this disease has a negative impact on people’s lives due to its chronicity, recurrence, pain, and impact on body image. For that reason, the important of applying specific questionnaires to better understand the impact of this disease on patients with DSC.

In conclusion, our study shows that early diagnosis and proper treatment could prevent disease progression, avoid cicatricial alopecia, improve inflammatory lesions, and help to reduce the impact on QoL in these patients.

## Financial support

None declared.

## Authors’ contributions

Paula Gerlero: Contributed to the study concept and design, the acquisition, analysis, and interpretation of data; Contributed to writing the manuscript and giving a critical review of important intellectual content; Participation in research guidance, intellectual participation in the therapeutic management of the cases studied and in the critical review of the literature; Approval of the final version of the manuscript.

Isabela Peron: Contributed to the study concept and design, the acquisition, analysis, and interpretation of data; Approval of the final version of the manuscript.

Isabella Doche: Contributed to the study concept and design, the acquisition, analysis, and interpretation of data; Participated in research guidance, intellectual participation in the therapeutic management of the cases studied and in the critical review of the literature; Approval of the final version of the manuscript.

Evelyn Freitas Rodrigues: Contributed to the study concept and design, the acquisition, analysis, and interpretation of data; Approval of the final version of the manuscript.

Thalita Macedo: Contributed to the study concept and design, the acquisition, analysis, and interpretation of data; Approval of the final version of the manuscript.

Maria Cecília Rivitti-Machado: Contributed to the study concept and design, the acquisition, analysis, and interpretation of data, contributed to writing the manuscript and giving a critical review of important intellectual content; participation in research guidance, intellectual participation in the therapeutic management of the cases studied and in the critical review of the literature; approval of the final version of the manuscript.

## Conflicts of interest

The authors Paula Gerlero, Isabela Peron, Thalita Macedo and Evelyn Freitas Rodrigues have no conflicts of interest to declare.

Isabella Doche is board of directors from the American Hair Research Society (2020‒2024).

Maria Cecilia Machado-Rivitti has conflicts of interest with: Abbvie, Janssen, Novartis, Boehringer-Ingelheim, Pfizer, Sanofi, UCB, America’s Health Foundation and Mantecorp.
